# Ginsenoside Rc Ameliorates Endothelial Insulin Resistance via Upregulation of Angiotensin-Converting Enzyme 2

**DOI:** 10.3389/fphar.2021.620524

**Published:** 2021-02-23

**Authors:** Yaozhen Wang, Wenwen Fu, Yan Xue, Zeyuan Lu, Yuangeng Li, Ping Yu, Xiaofeng Yu, Huali Xu, Dayun Sui

**Affiliations:** ^1^ Department of Pharmacology, School of Pharmaceutical Sciences, Jilin University, Changchun, China; ^2^ Department of Burn Surgery, The First Hospital of Jilin University, Changchun, China

**Keywords:** ginsenoside Rc, endothelial cells, insulin resistance, endothelial dysfunction, ACE2, MLN-4760

## Abstract

Type 2 diabetes mellitus (T2DM) is a major health concern which may cause cardiovascular complications. Insulin resistance (IR), regarded as a hallmark of T2DM, is characterized by endothelial dysfunction. Ginsenoside Rc is one of the main protopanaxadiol-type saponins with relatively less research on it. Despite researches confirming the potent anti-inflammatory and antioxidant activities of ginsenoside Rc, the potential benefits of ginsenoside Rc against vascular complications have not been explored. In the present study, we investigated the effects of ginsenoside Rc on endothelial IR and endothelial dysfunction with its underlying mechanisms using high glucose- (HG-) cultured human umbilical vein endothelial cells (HUVECs) *in vitro* and a type 2 diabetic model of db/db mice *in vivo*. The results showed that ginsenoside Rc corrected the imbalance of vasomotor factors, reduced the production of Ang (angiotensin) II, and activated angiotensin-converting enzyme 2 (ACE2)/Ang-(1–7)/Mas axis in HG-treated HUVECs. Besides, ginsenoside Rc improved the impaired insulin signaling pathway and repressed oxidative stress and inflammatory pathways which constitute key factors leading to IR. Interestingly, the effects of ginsenoside Rc on HG-induced HUVECs were abolished by the selective ACE2 inhibitor MLN-4760. Furthermore, ginsenoside Rc exhibited anti-inflammatory as well as antioxidant properties and ameliorated endothelial dysfunction via upregulation of ACE2 in db/db mice, which were confirmed by the application of MLN-4760. In conclusion, our findings reveal a novel action of ginsenoside Rc and demonstrate that ginsenoside Rc ameliorated endothelial IR and endothelial dysfunction, at least in part, via upregulation of ACE2 and holds promise for the treatment of diabetic vascular complications.

## Introduction

Insulin resistance (IR) is usually defined as decreased sensitivity to metabolic actions of insulin in target tissues including muscle, liver, and adipose (Kim et al., 2006; Kong et al., 2019) and plays a critical role in the development of type 2 diabetes mellitus (T2DM), which is frequently present in obesity, dyslipidemia, and metabolic syndrome (DeFronzo and Ferrannini, 1991; Petersen et al., 2007). In addition to its essential metabolic actions, insulin has important vascular actions mediated by endothelium via insulin signaling pathways (Baron and Clark, 1997). IR is intimately associated with endothelial dysfunction characterized by reduced NO bioavailability, increased oxidative stress, elevated expression of proinflammatory factors, and abnormal vasoreactivity (Barac et al., 2007). Not only does IR contribute to endothelial dysfunction (Muniyappa and Sowers, 2013), but there are also reciprocal relationships between IR and endothelial dysfunction (Kim et al., 2006), both of which play a major role in the development of cardiovascular complications (Bonetti et al., 2003; Huang, 2009; Ko et al., 2010; Muris et al., 2012). Consequently, intervention aiming to protect against endothelial IR is critical for the prevention and improvement of diabetic vascular complications.

Angiotensin (Ang) converting enzyme 2 (ACE2), a homolog of ACE, is a monocarboxypeptidase which cleaves Ang II to generate Ang-(1–7) and is mainly expressed in vascular endothelial cells and the renal tubular epithelium (Donoghue et al., 2000; Tipnis et al., 2000). Although ACE2 share a homology of 42% with ACE, it is nonsensitive to ACE inhibitors and has a 400-fold higher affinity for Ang II than Ang I (Donoghue et al., 2000; Tipnis et al., 2000). Ang-(1–7), the most important active product of ACE2 (Vickers et al., 2002; Jiang et al., 2014), interacts with its receptor Mas which is a G protein-coupled receptor to exert actions including vasodilation, antihypertrophy, anti-inflammation, antioxidation, and improving vascular endothelial dysfunction (Santos et al., 2003; Rabelo et al., 2008; Gwathmey et al., 2010). Thus, the ACE2/Ang-(1–7)/Mas axis has been proposed to counteract the classical renin-angiotensin system (RAS) and ACE/Ang II/angiotensin II type 1 receptor (AT_1_R) axis in the cardiovascular system and kidney (Crackower et al., 2002; Santos et al., 2003; Mercure et al., 2008; Oudit et al., 2010). It has already been reported that ACE2/Ang-(1–7)/Mas axis could ameliorate metabolic IR (Liu et al., 2012; Cao et al., 2014; Echeverría-Rodríguez et al., 2014). Although Ang-(1–7) has been reported to improve endothelial dysfunction in db/db mice and Ang II-induced IR in endothelial cells (Tassone et al., 2013; Zhang et al., 2015), literature about ACE2/Ang-(1–7)/Mas axis emphasizing endothelial IR remains scarce.

Panax ginseng is one of the most well-known and commonly used traditional herb medicines in oriental countries, which has long been used for treating cardiovascular disorders, cancer, and diabetes (Zhou et al., 2004; Kim, 2012). Ginsenosides are the major bioactive components of ginseng and responsible for the majority of its pharmacological properties (Lü et al., 2009). Ginsenoside Rc is one of the major protopanaxadiol-type saponins with relatively less research on it and has been demonstrated to exhibit potent anti-inflammatory activities both *in vitro* and *in vivo* (Yu et al., 2016; Yu et al., 2017). Furthermore, ginsenoside Rc has been suggested to show antioxidant effects involved in scavenging reactive oxygen species (ROS) (Gillis, 1997; Kim et al., 2014; Oh et al., 2017). However, the potential benefits of ginsenoside Rc against vascular complications have not been explored. Considering the well-established role of inflammation and oxidative stress in IR (Kim et al., 2006; McNelis and Olefsky, 2014) as well as the essential part played by ACE2/Ang-(1–7)/Mas axis in cardiovascular complications (Jiang et al., 2014; Patel et al., 2014), we wondered whether ginsenoside Rc could contribute to the alleviation of endothelial IR and endothelial dysfunction via activating ACE2/Ang-(1–7)/Mas axis.

In the present study, we aimed to investigate the effects of ginsenoside Rc on endothelial IR with its underlying mechanisms *in vitro* and *in vivo*.

## Materials and Methods

### Reagents

Ginsenoside Rc (purity＞98%) was provided by Professor Yifa Zhou (School of life sciences, Northeast Normal University, China). D-(+)-glucose was obtained from Sigma (St. Louis, MO, United States). Human umbilical vein endothelial cells (HUVECs) were purchased from Procell (Wuhan, China). Ham’s F-12K complete medium (including 100 ug/ml heparin, 50 ug/ml endothelial cell growth supplement, 10% fetal bovine serum, and 1% penicillin/streptomycin) and recombinant human insulin were purchased from Procell (Wuhan, China). MLN-4760 was purchased from Merck KGaA (Darmstadt, Germany). Nitric Oxide (NO) assay kit, total cholesterol (TC), triglyceride (TG), low-density lipoprotein cholesterol (LDL-C), high-density lipoprotein cholesterol (HDL-C), and ELISA kits for insulin, TNF-α, IL-6, Ang II, and Ang-(1–7) were purchased from Nanjing Jiancheng Biotechnology (Nanjing, China). The BCA Protein Assay Kit, ROS Assay Kit, Alexa Fluor 488-Labeled Goat Anti-Rabbit IgG, Cy3-labeled Goat Anti-Rabbit IgG, and 4′,6-diamidino-2-phenylindole (DAPI) were purchased from Beyotime Biotechnology (Shanghai, China). Primary antibodies against p-IRS1^Ser307^, IRS1, PI3K p-85, p-Akt^Ser473^, Akt, p-eNOS^Ser1177^, eNOS, p-IKKβ^Ser177/181^, p-IKKβ, p-JNK^Thr183/Tyr185^, and JNK were purchased from Cell Signaling Technology (Danvers, MA, United States). Primary antibodies against ACE2, Mas, p-IRS1^Tyr896^, p-PI3K p85^Tyr607^, NOX2, NOX4, and p-NF-κB p65^Ser536^ were purchased from Abcam (Cambridge, United Kingdom). The anti-GAPDH primary antibody was purchased from ZSGB Biotechnology (Beijing, China). The anti-rabbit and anti-mouse secondary antibodies were purchased from Cell Signaling Technology (Danvers, MA, United States).

### Human Umbilical Vein Endothelial Cells Culture and Treatment

HUVECs were cultured in Ham’s F-12K complete medium at 37°C in a humidified atmosphere of 5% CO_2_. To establish the endothelial IR model *in vitro*, HUVECs were cultivated in Ham’s F-12K complete medium supplemented with 23 mM glucose (7 mM for control) for 24 h. For drug treatment, cells were incubated with a dose range of ginsenoside Rc (0, 1, 10, 20, 50, and 100 μM) for 24 h. Some cells were treated with normal glucose (NG, 7 mM) with or without ginsenoside Rc (50 μM) and high glucose (HG, 30 mM) with or without ginsenoside Rc (25 and 50 μM) for 24 h followed by incubation with 100 nM insulin for 30 min. Some cells were exposed to NG, HG with or without MLN-4760 (100 nM), a selective ACE2 inhibitor, and HG plus ginsenoside Rc (50 μM) with or without MLN-4760 (100 nM) followed by incubation with 100 nM insulin for 30 min.

### Cell Viability Study

To determine the cytotoxicity and nontoxic concentration of ginsenoside Rc, cell viability was measured by MTT (3-(4,5-dimethyl-2-thiazolyl)-2,5-diphenyl-2-H-tetrazolium bromide) (Sigma, St. Louis, MO, United States) assay as described previously (Zhang et al., 2018). In brief, cells were seeded in 96-well plates and after treatment, sterile MTT (5 mg/ml in PBS) solution was added to each well, and plates were then incubated at 37°C for 4 h, after which the supernatant was discarded and formazan was dissolved in DMSO. Then the plates were shaken for 10 min and absorbance was read at 570 nm with a microplate reader (SpectraMax Plus384, Molecular Devices, United States). The percentage of survival was calculated as a fraction of the negative control.

### Biochemical Analysis of Culture Medium

After treatment, culture medium was collected. The NO concentration was detected based on the nitrate reductase method according to the manufacturer’s instructions. Levels of Ang II and Ang-(1–7) in the supernatant were assessed using ELISA kits according to the manufacturer’s specifications.

### Measurement of Intracellular ROS

Intracellular ROS generation was monitored by 2′,7′-dichlorofluorescein diacetate (DCFH-DA), which was not fluorescent per se, but it readily diffused into cells and was hydrolyzed by nonspecific esterases to yield nonpermeable 2′,7′-dichlorofluorescein (DCFH) which was subsequently oxidized to the highly fluorescent compound dichlorofluorescein (DCF) (Wang and Joseph, 1999). Thus, fluorescence intensity was proportional to the amount of ROS produced in the cells. After treatment, HUVECs were washed with PBS and then incubated with 10 μM DCFH-DA at 37°C for 20 min, after which cells were washed with PBS three times and examined under a Nikon TE-2000U inverted fluorescence microscope (magnification, ×100).

### Quantitative Real-Time PCR

Total RNA was isolated using Trizol reagent (Invitrogen, Carlsbad, CA) according to the manufacturer’s instructions. RNA was quantified by optical density measurements at 260 and 280 nm, after which cDNA was synthesized from 1 ug of total RNA using a TransScript® All-in-One First-Strand cDNA Synthesis SuperMix for qPCR (TransGen Biotech, Beijing, China). qPCR was performed using a TransStart® Tip Green qPCR SuperMix (TransGen Biotech, Beijing, China) on an Agilent StrataGene Mx3000P (Santa Clara, CA, United States). The results were analyzed using the 2^−ΔΔCt^ method. The relative gene expression was normalized to GAPDH. The sequences of the primers used were as follows: ACE2 (human) forward: 5′-ACA​GCC​AAC​ACT​TGG​ACC​TC-3′, reverse: 5′-AGG​AGG​TCT​GAA​CAT​CAT​CAG​TG-3'; Mas (human) forward: 5′-GCG​TCC​CAG​TTT​TTC​ATT​GCT-3′, reverse: 5′-TTC​TCA​TCC​GGA​AGC​ACA​GG-3'; ET-1 (human) forward: 5′-AAC​CGA​GCA​CAT​TGG​TGA​CA-3′, reverse: 5′-TCC​TTT​GCC​AGT​CAG​GAA​CC-3'; TNF-α (human) forward: 5′-AGA​ACT​CAC​TGG​GGC​CTA​CA-3′, reverse: 5′-GCT​CCG​TGT​CTC​AAG​GAA​GT-3'; IL-1β (human) forward: 5′-CTT​CGA​GGC​ACA​AGG​CAC​AA-3′, reverse: 5′-TTC​ACT​GGC​GAG​CTC​AGG​TA-3'; IL-6 (human) forward: 5′-TCA​ATG​AGG​AGA​CTT​GCC​TG-3′, reverse: 5′-GAT​GAG​TTG​TCA​TGT​CCT​GC-3'; GAPDH (human) forward: 5′-AGA​AGG​CTG​GGG​CTC​ATT​TG-3′, reverse: 5′-AGG​GGC​CAT​CCA​CAG​TCT​TC-3'.

### Immunofluorescence (IF)

After treatment, HUVECs cultured on coverslips in 24-well plates were washed with PBS, fixed with 4% paraformaldehyde for 15 min, permeabilized with 0.3% Triton X-100 for 10 min, and blocked with 5% normal goat serum at room temperature for 1 h. Cells were then incubated with primary antibodies against ACE2, NOX2, and p-NF-κB p65^Ser536^ overnight at 4°C, after which cells were washed with PBS with subsequent incubation with appropriate fluorescent secondary antibodies at room temperature for 1 h. Then cells were stained with DAPI for 5 min and images were captured by a Nikon TE-2000U inverted fluorescence microscope (magnification, ×200).

### Animals and Treatments

Male C57BLKS/J, leptin receptor-deficient db/db mice, and littermate wild type (WT) m/m mice (8 weeks old) were supplied by the Model Animal Research Center of Nanjing University. All mice were kept at 22–24°C with a 12 h light/dark cycle and free access to diet and water. WT mice were randomly divided into two groups to receive either distilled water or ginsenoside Rc (20 mg/kg/d, dissolved in sterile distilled water, intragastric [ig.] administration) for 4 weeks. The db/db mice were randomized to receive distilled water (ig. administration) with or without MLN-4760 (0.5 mg/kg/d, intraperitoneal [ip.] administration) and ginsenoside Rc (20 mg/kg/d, ig. administration) with or without MLN-4760 (0.5 mg/kg/d, ip. administration) (Ye et al., 2012; Lin et al., 2017).

### Fasting Blood Glucose Measurement

Throughout the 4-week treatment period, fast blood glucose (FBG) was monitored weekly on lateral tail vein blood samples using a Blood Glucose Test Meter (GlucoLab, Infopia Co., Ltd., Anyang, Kyunggi, Korea).

### Biochemical Analysis of Serum and Aorta Tissue Homogenate

Blood samples were drawn after 4-week treatment. The collected blood samples were clotted for 1 h at room temperature and subsequently centrifuged at 2500 rpm for 15 min. The supernatant serum was collected and stored at −80°C. The tissues of aortas were excised and homogenized with ice-cold saline in the homogenizer. The homogenate was centrifuged at 3000 rpm for 10 min and the supernatant was collected and stored at −80°C. Serum TC, TG, LDL-C, HDL-C, insulin, TNF-α, IL-6, Ang II, Ang-(1–7), and aorta Ang II as well as Ang-(1–7) concentrations were determined using diagnostic kits according to the manufacturer’s instructions.

### Thoracic Aortic Ring Relaxation Assay

Vasodilation function was assessed *ex vivo* in a wire myograph (Danish Myo Technology) as previously described (Lan et al., 2011; Watt et al., 2017). The thoracic aortic rings (3 mm) from anesthetized mice, free of adipose and connective tissues, were mounted in an organ bath containing Krebs solution (mM: NaCl 118, KCl 4.7, NaHCO_3_ 25, MgCl_2_ 1.2, CaCl_2_ 2.5, KH_2_PO_4_ 1.2, glucose 11, and pH 7.4) gassed with 95% O_2_/5% CO_2_ at 37°C. Rings were equilibrated at a resting tension of 2.0 g for 90 min, after which 60 mM KCl was added to the organ bath to evaluate the viability of the ring preparation by assessing constriction of the rings before each experiment. Relaxation responses to cumulative addition of acetylcholine (ACh, 1 nM-10 μM) and sodium nitroprusside (SNP, 1 nM-10 μM) were performed after vasoconstriction curves of rings preconstricted with phenylephrine (PE, 1 μM) reached the plateau phase. Relaxation responses are expressed as % decrement in preconstricted tension.

### Immunohistochemistry (IHC)

The aortas were dissected and fixed in 4% paraformaldehyde. Then the aortas were embedded in paraffin and sections of the aortic rings were sliced, dewaxed, hydrated, washed with PBS three times for 5 min each time, and treated with 3% H_2_O_2_ for 10 min to eliminate endogenous peroxidase with subsequent antigen retrieval for 15 min and blocking with 5% normal goat serum for 20 min at room temperature. For immunohistochemical studies, sections were incubated with appropriate primary antibodies at 4°C overnight followed by incubation with the biotin-labeled secondary antibody for 30 min at room temperature. Chromogenic reactions were mediated by SP (streptavidin-peroxidase) and DAB (3,3-N-diaminobenzidine tetrahydrochloride), after which sections were washed and counterstained with hematoxylin. All stained sections were viewed and photographed by using a light microscope at a magnification of 400× (BX51, Olympus, Japan). Positive areas were quantified with Motic Images Advanced 3.2.

### Western Blot Analysis

Cells with different treatment were harvested and lysed in radioimmunoprecipitation assay (RIPA) lysis buffer containing phenylmethanesulfonyl fluoride (PMSF) on ice. The aorta tissues were homogenized on ice in RIPA lysis buffer containing PMSF. The lysate was centrifuged at 12,000 g at 4°C for 10 min and the protein concentrations were determined by BCA assay. The protein extraction was loaded onto 12% polyacrylamide-SDS gel. After electrophoresis, the gel was electrotransferred onto a polyvinylidene fluoride (PVDF) membrane which was subsequently blocked with 5% nonfat milk for 1 h at room temperature. Then, the membrane was incubated in appropriate primary antibodies at 4°C overnight. The next day after secondary antibody incubation, the PVDF membrane was visualized by using ECL chemiluminescence. GAPDH was used as an internal control. The results were expressed by grayscale value analyzed in NIH ImageJ software.

### Statistical Analysis

All results were expressed as mean ± SD. Statistical differences were evaluated by one-way ANOVA followed by Tukey’s test. *p* < 0.05 was considered to be significant.

## Results

### Ginsenoside Rc Improved the Imbalance of Vasomotor Factors and Inhibited ROS Production and Inflammatory Responses Without Affecting Cell Viability of HUVECs

**FIGURE 1:**
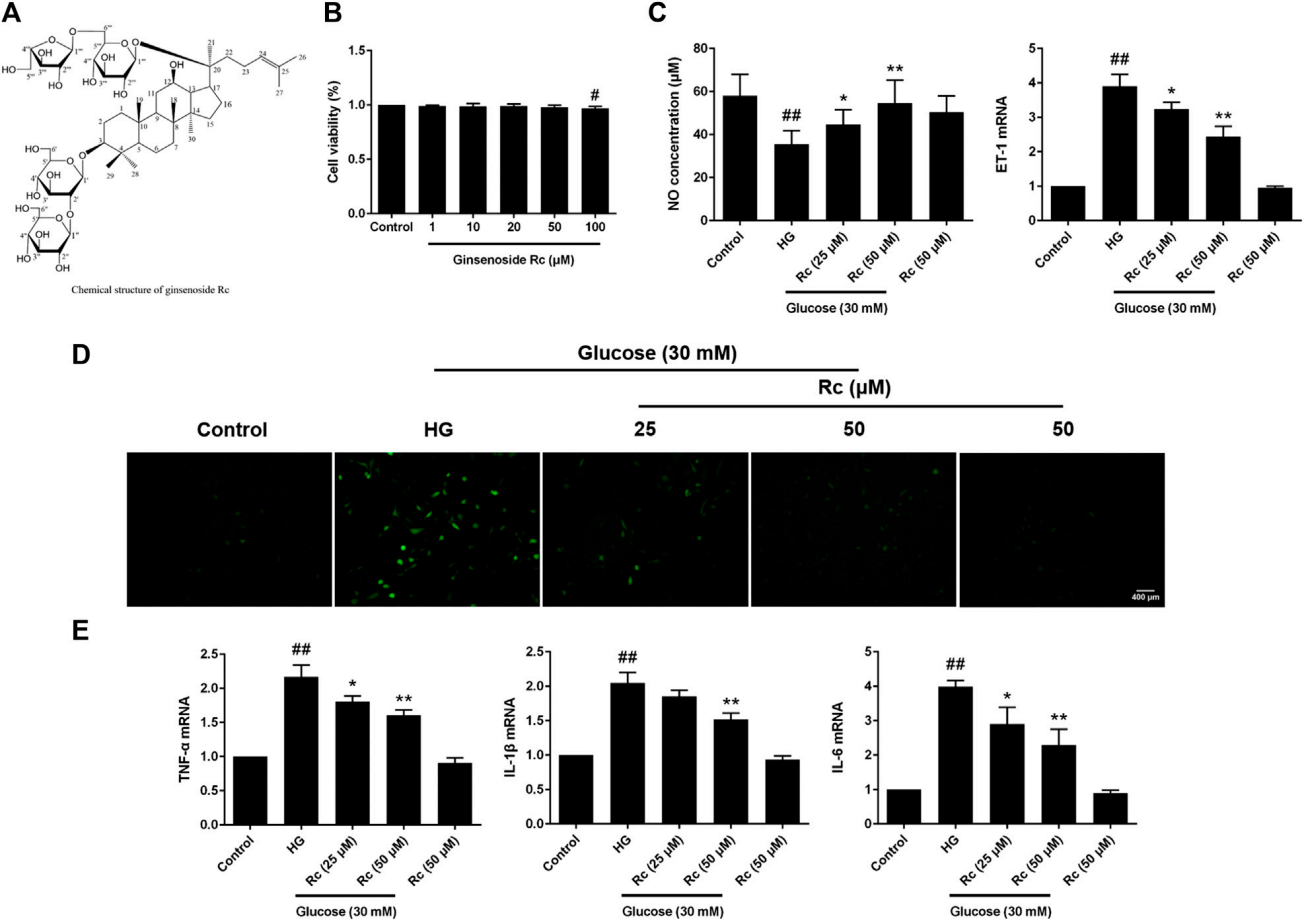
Effects of ginsenoside Rc on the production of NO, level of ET-1 mRNA, intracellular ROS, and proinflammatory cytokine mRNA levels in HUVECs. The cells were treated with increasing concentrations of ginsenoside Rc (0, 1, 10, 20, 50, and 100 μM) for 24 h and some cells were treated with HG, with or without ginsenoside Rc (25 and 50 μM), for 24 h followed by insulin (100 nM) stimulation for 30 min. **(A)** The chemical structure of ginsenoside Rc. **(B)** Cell viability. **(C)** The production of NO in the culture medium and ET-1 mRNA level. **(D)** Intracellular ROS. **(E)** TNF-α, IL-1β, and IL-6 mRNA levels. Magnification, ×100; scale bar = 400 μm. Data were expressed as mean ± SD from three independent experiments. ^#^ *p* < 0.05 and ^##^ *p* < 0.01 compared with control and ^*^ *p* < 0.05 and **p < 0.01 compared with HG.

We first detected the cytotoxicity of ginsenoside Rc in HUVECs and found that it did not affect cell viability until the concentration reached 100 μM (Figure 1B). As NO and ET-1 secreted by endothelium which is also an important endocrinal organ are often considered the markers of endothelial IR (Kearney et al., 2008; Baratchi et al., 2017), we investigated the NO content in the culture medium and ET-1 mRNA. As was shown in Figure 1C, HG induced a significant decrease of NO as well as a remarkable augmentation of ET-1 mRNA in HUVECs, which were improved by ginsenoside Rc in a dose-dependent manner without affecting cells with NG treatment. We next evaluated the effects of ginsenoside Rc on HG-induced ROS and proinflammatory factors. As was shown in Figures 1D,E, HG significantly increased intracellular ROS and mRNA levels of TNF-α, IL-1β, and IL-6; however, an obvious reduction of ROS and these cytokines were observed in the HG groups treated with ginsenoside Rc. Ginsenoside Rc alone did not influence ROS and proinflammatory cytokines.

### Ginsenoside Rc Reduced the Production of Ang II and Activated ACE2/Ang-(1–7)/Mas Axis in HUVECs

**FIGURE 2:**
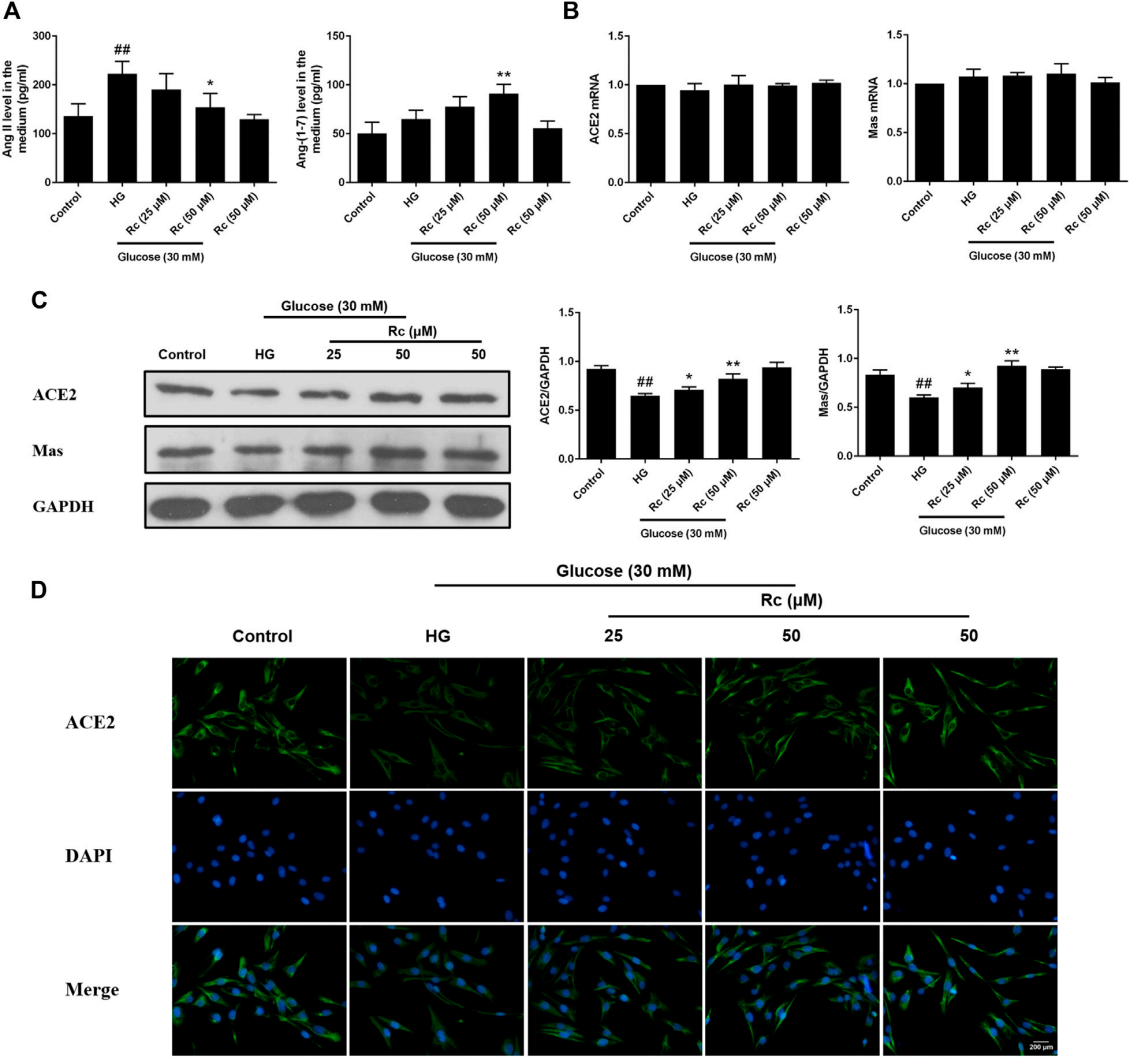
Effects of ginsenoside Rc on Ang II level and ACE2/Ang-(1–7)/Mas axis in HUVECs. The cells were treated with HG, with or without ginsenoside Rc (25 and 50 μM) for 24 h followed by insulin (100 nM) stimulation for 30 min. **(A)** The production of Ang II and Ang-(1–7) in the culture medium. **(B)** ACE2 and Mas mRNA levels. **(C)** Western blot analysis for ACE2 and Mas. **(D)** IF staining for ACE2. Magnification, ×200; scale bar = 200 μm. Data were expressed as mean ± SD from three independent experiments. ^##^ *p* < 0.01 compared with control and ^*^ *p* < 0.05 and ^**^ *p* < 0.01 compared with HG.

To determine the role of ACE2/Ang-(1–7)/Mas axis in endothelial IR induced by HG with effects of ginsenoside Rc on it, we examined the production of Ang II and Ang-(1–7) in the culture medium and the expressions of ACE2 and Mas at protein and mRNA level. As was shown in Figure 2A, HG remarkably increased the production of Ang II but induced a slight increase in Ang-(1–7) without significance; however, ginsenoside Rc significantly reduced Ang II production and further increased Ang-(1–7) content in a dose-dependent manner. Immunoblot analysis revealed that ginsenoside Rc markedly attenuated the reduction of ACE2 and Mas protein expressions induced by HG, which was also confirmed by IF for ACE2 (Figures 2C,D). Interestingly, there was no significant change in mRNA levels of ACE2 and Mas among each group (Figure 2B). Ginsenoside Rc alone did not affect the above indexes. These results indicate that ginsenoside Rc is capable of reducing Ang II and activating ACE2/Ang-(1–7)/Mas axis in HG-induced HUVECs of IR.

### Ginsenoside Rc Improved the Impaired Insulin Signaling Pathway in HUVECs

**FIGURE 3:**
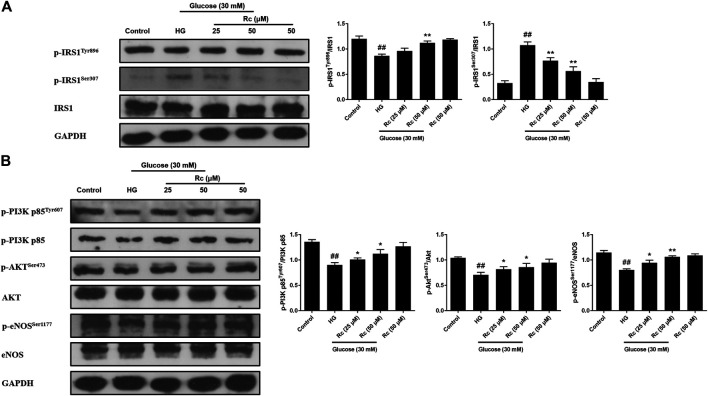
Effects of ginsenoside Rc on the insulin signaling pathway in HUVECs. The cells were treated with HG, with or without ginsenoside Rc (25 and 50 μM), for 24 h followed by insulin (100 nM) stimulation for 30 min. **(A)** Western blot analysis for p-IRS1^Tyr896^ and p-IRS1^ser307^. **(B)** Western blot analysis for p-PI3K p85^Tyr607^, p-Akt^Ser473^, and p-eNOS^Ser1177^. Data were expressed as mean ± SD from three independent experiments. ^##^ *p* < 0.01 compared with control and ^*^ *p* < 0.05 and ^**^ *p* < 0.01 compared with HG.

To address whether ginsenoside Rc did improve IR in HUVECs, we examined the insulin signaling pathway using Western blotting. IR is an abnormal status attributed to certain factors and we have found that HG induced IR in HUVECs without affecting cell viability (data not shown). It is plausible that just the state of cells has changed. The pathway consisting of IRS-1/PI3K/Akt/eNOS was dramatically impaired after HG exposure. Phosphorylation of IRS-1 serine, a key event linking inflammation and oxidative stress to impaired insulin signaling, displayed a significant increase, while a remarkable decrease of the phosphorylation of tyrosine for activation was observed. In addition, phosphorylation of PI3K/Akt/eNOS was also reduced significantly. However, there was a tendency to gradually recover in the insulin signaling benefiting from ginsenoside Rc treatment without affecting NG-treated cells (Figures 3A,B). These findings strongly suggest that ginsenoside Rc could alleviate IR in HG-induced HUVECs.

### Ginsenoside Rc Reduced the Expression of NOX2 in HUVECs

**FIGURE 4:**
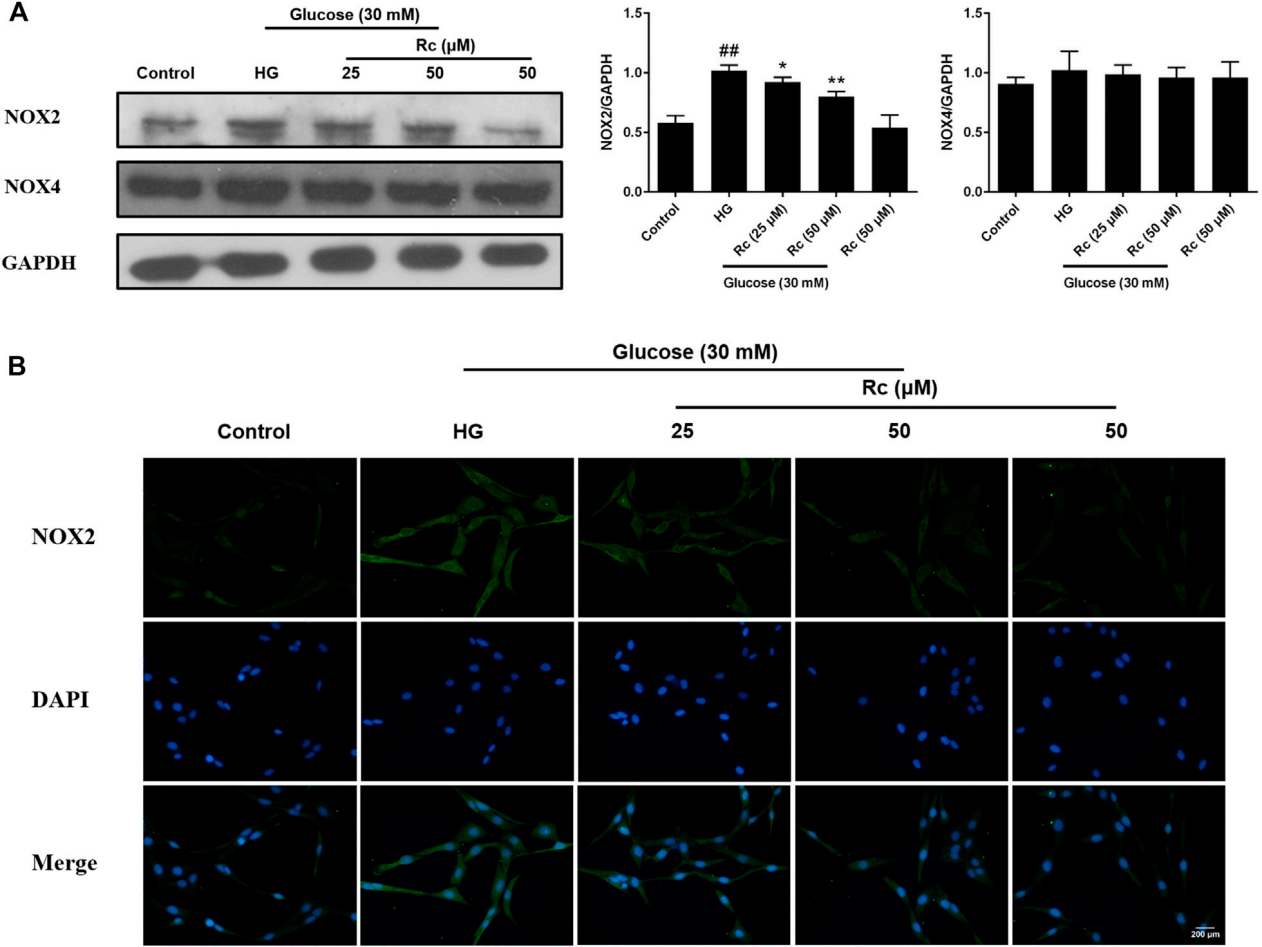
Effects of ginsenoside Rc on the protein expression of NADPH oxidase in HUVECs. The cells were treated with HG, with or without ginsenoside Rc (25 and 50 μM), for 24 h followed by insulin (100 nM) stimulation for 30 min. **(A)** Western blot analysis for NOX2 and NOX4. **(B)** IF staining for NOX2. Magnification, ×200; scale bar = 200 μm. Data were expressed as mean ± SD from three independent experiments. ^##^ *p* < 0.01 compared with control and ^*^ *p* < 0.05 and ^**^ *p* < 0.01 compared with HG.

NADPH oxidase has been considered a major source of ROS in blood vessels, which is also closely related to IR (Delbosc et al., 2005; Wei et al., 2006; Madrigal-Matute et al., 2015). We detected protein expressions of NOX2 and NOX4, two major components of the NOX family which serves as the catalytic subunits of NADPH oxidase. As was shown in Figure 4A, treatment with HG presented a significant augmentation in the protein expression of NOX2, while ginsenoside Rc markedly suppressed NOX2 expression concentration dependently without affecting NG-treated cells, which was also confirmed by IF for NOX2 (Figure 4B). Simultaneously, HG exposure failed to induce a remarkable increase in the NOX4 expression and there was no significant change among each group. These results clearly demonstrate that NOX2 inhibition is implicated in the effects of ginsenoside Rc on IR improvement.

### Ginsenoside Rc Inhibited Inflammatory Pathways Related to Abnormal Phosphorylation of IRS-1 in HUVECs

**FIGURE 5:**
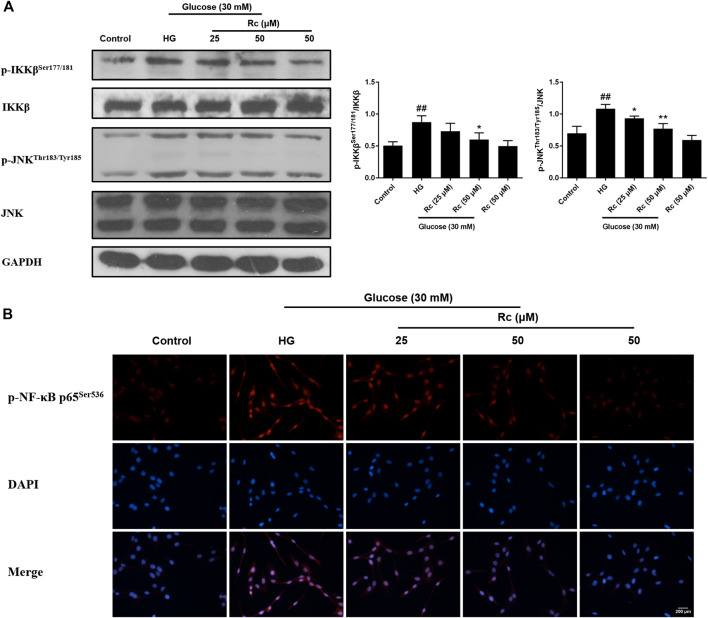
Effects of ginsenoside Rc on p-IKKβ^Ser177/181^ and p-JNK^Thr183/Tyr185^ phosphorylation in HUVECs followed by insulin (100 nM) stimulation for 30 min. The cells were treated with HG, with or without ginsenoside Rc (25 and 50 μM), for 24 h. **(A)** Western blot analysis for p-IKKβ^Ser177/181^ and p-JNK^Thr183/Tyr185^. **(B)** IF staining for p-NF-κB p65^Ser536^. Magnification, ×200; scale bar = 200 μm. Data were expressed as mean ± SD from three independent experiments. ^##^ *p* < 0.01 compared with control and ^*^ *p* < 0.05 and ^**^ *p* < 0.01 compared with HG.

Studies have validated the key roles of IKKβ and JNK in the inflammatory signaling as well as their actions on phosphorylating inhibitory sites of IRS (Ozes et al., 2001; Gao et al., 2002). To further demonstrate the anti-inflammatory properties of ginsenoside Rc with its effects on ameliorating IR, we examined IKKβ/NF-κB activation and JNK phosphorylation. As was shown in Figure 5A, HG resulted in enhanced phosphorylation of IKKβ and JNK, which was significantly inhibited by ginsenoside Rc in a dose-dependent manner without affecting NG-treated cells. IF analysis also showed that ginsenoside Rc remarkably suppressed the nuclear translocation of p-NF-κB p65 in HG-induced HUVECs (Figure 5B). Taken together, these results support that ginsenoside Rc effectively inhibits inflammatory pathways related to abnormal phosphorylation of IRS-1.

### Upregulation of ACE2 Was Associated With the Effects of Ginsenoside Rc on Improving IR in HUVECs

**FIGURE 6:**
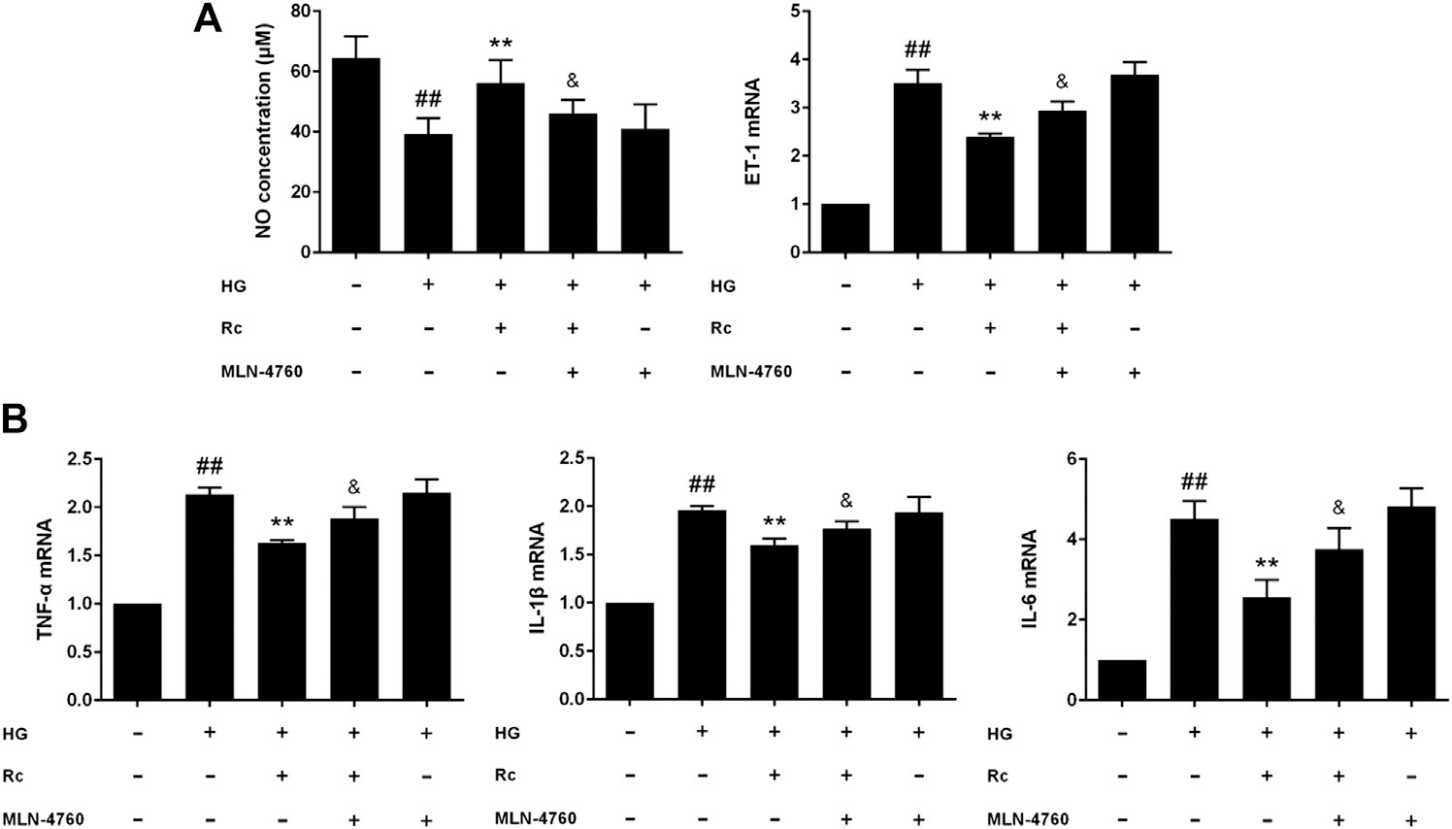
Effects of ginsenoside Rc with MLN-4760 on the production of NO, level of ET-1, and proinflammatory cytokine mRNA levels in HUVECs followed by insulin (100 nM) stimulation for 30 min. The cells were treated with HG plus ginsenoside Rc (50 μM) with or without MLN-4760 (100 nM) for 24 h. **(A)** The production of NO in the culture medium and ET-1 mRNA level. **(B)** TNF-α, IL-1β, and IL-6 mRNA levels. Data were expressed as mean ± SD from three independent experiments. ^##^ *p* < 0.01 compared with control, ^**^ *p* < 0.01 compared with HG, and ^&^ *p* < 0.05 compared with HG **+** ginsenoside Rc.

**FIGURE 7:**
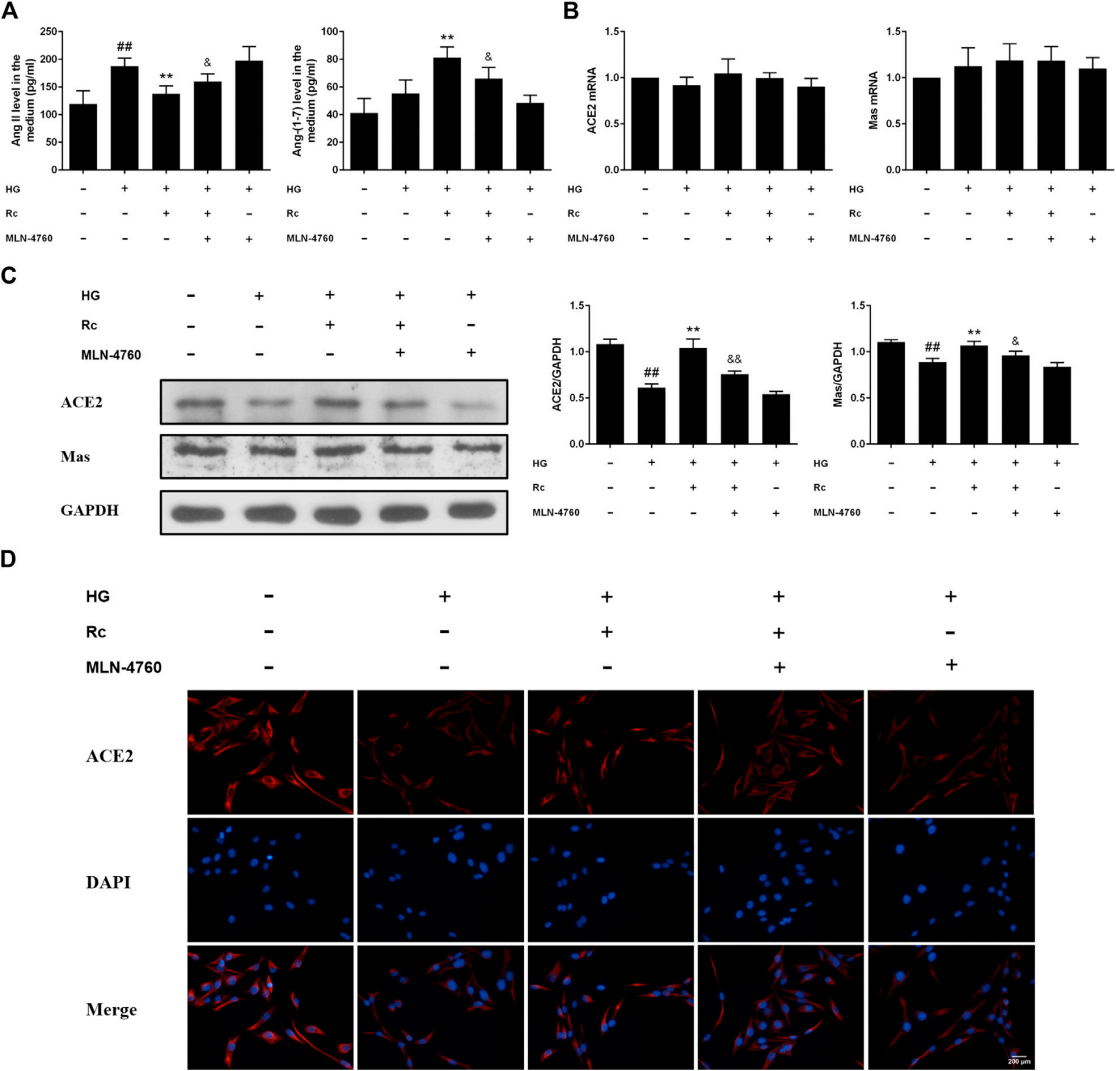
Effects of ginsenoside Rc with MLN-4760 on Ang II level and ACE2/Ang-(1–7)/Mas axis in HUVECs followed by insulin (100 nM) stimulation for 30 min. The cells were treated with HG plus ginsenoside Rc (50 μM) with or without MLN-4760 (100 nM) for 24 h. **(A)** The production of Ang II and Ang-(1–7) in the culture medium. **(B)** ACE2 and Mas mRNA levels **(C)** Western blot analysis for ACE2 and Mas. **(D)** IF staining for ACE2. Magnification, ×200; scale bar = 200 μm. Data were expressed as mean ± SD from three independent experiments. ^##^ *p* < 0.01 compared with control, ^**^ *p* < 0.01 compared with HG, and ^&^ *p* < 0.05 and ^&&^ *p* < 0.01 compared with HG **+** ginsenoside Rc.

**FIGURE 8:**
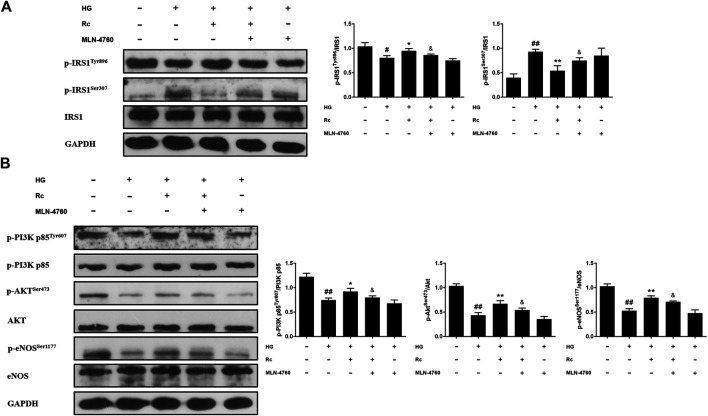
Effects of ginsenoside Rc with MLN-4760 on the insulin signaling pathway in HUVECs. The cells were treated with HG plus ginsenoside Rc (50 μM) with or without MLN-4760 (100 nM) for 24 h followed by insulin (100 nM) stimulation for 30 min. **(A)** Western blot analysis for p-IRS1^Tyr896^ and p-IRS1^ser307^. **(B)** Western blot analysis for p-PI3K p85^Tyr607^, p-Akt^Ser473^, and p-eNOS^Ser1177^. Data were expressed as mean ± SD from three independent experiments. ^#^ *p* < 0.05 and ^##^ *p* < 0.01 compared with control, ^*^ *p* < 0.05 and ^**^ *p* < 0.01 compared with HG, and ^&^ *p* < 0.05 compared with HG **+** ginsenoside Rc.

To further validate the role of ACE2 in the mechanism of ginsenoside Rc on improving IR in HG-induced HUVECs, we compared the effects of ginsenoside Rc with those of the selective ACE2 inhibitor MLN-4760. As was shown in Figure 6A, ginsenoside Rc-induced restoration of NO and reduction of ET-1 were significantly inhibited by MLN-4760. Similar results were observed in proinflammatory cytokines (Figure 6B). Additionally, MLN-4760 partially reversed the effects of ginsenoside Rc on reducing Ang II and augmenting Ang-(1–7) content (Figure 7A). Meanwhile, there was no remarkable difference among the relative mRNA level of each group (Figure 7B). Ginsenoside Rc-mediated upregulation of ACE2 and Mas protein expressions were also partially abrogated by MLN-4760 (Figures 7C,D). In the insulin signaling pathway, MLN-4760 partly abolished the effects of ginsenoside Rc on activating IRS-1/PI3K/Akt/eNOS (Figures 8A,B). Intriguingly, MLN-4760 alone did not exacerbate the impairment in this pathway in HG-treated cells. Together, these data provide compelling evidence that the effects of ginsenoside Rc on ameliorating HG-induced IR in HUVECs are dependent on upregulation of ACE2, at least in part.

### Inhibition of ACE2 by MLN-4760 Partly Abolished the Effects of Ginsenoside Rc on NOX2 Inhibition and Inflammation Pathways in HUVECs

**FIGURE 9:**
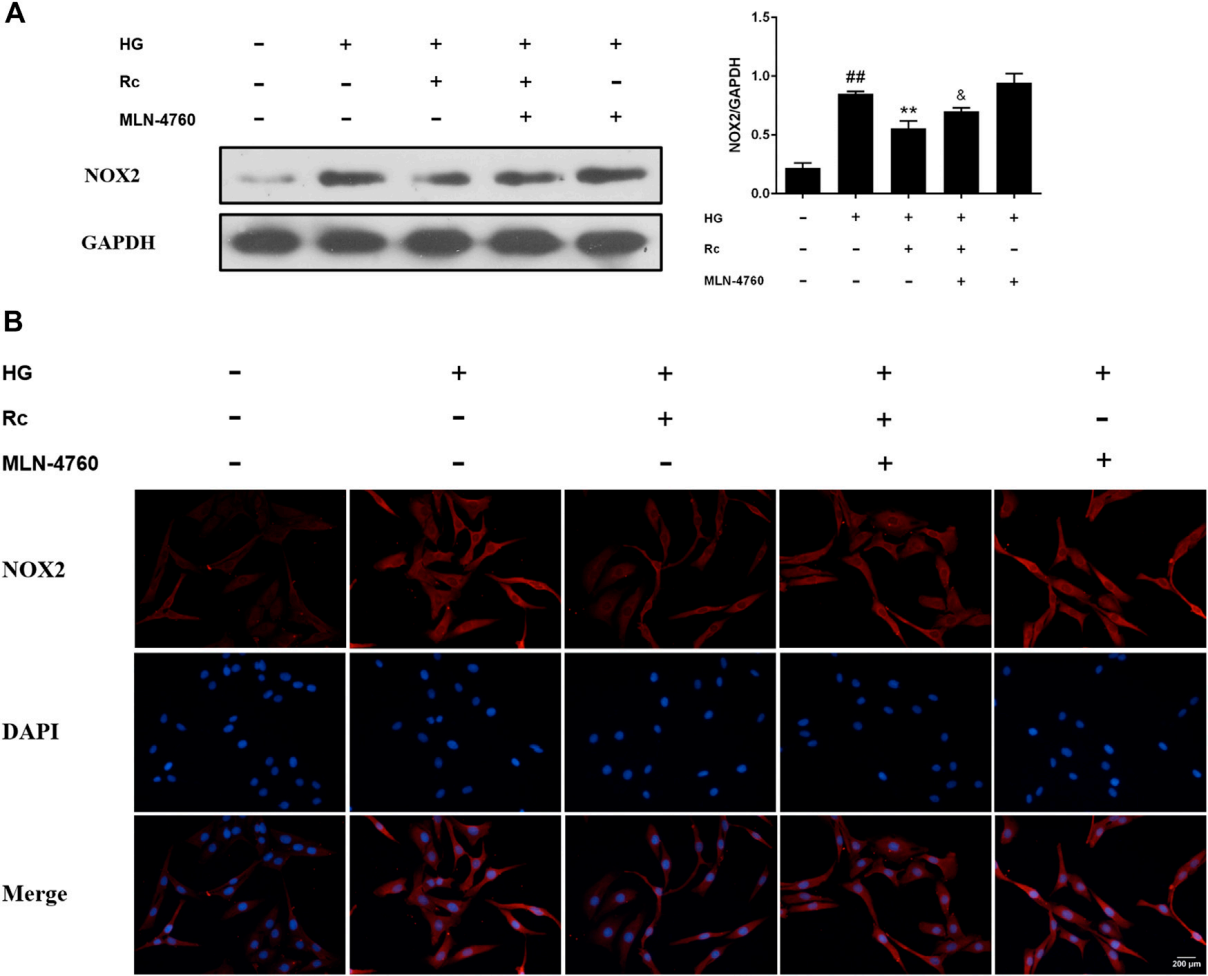
Effects of ginsenoside Rc with MLN-4760 on the protein expression of NOX2 in HUVECs followed by insulin (100 nM) stimulation for 30 min. The cells were treated with HG plus ginsenoside Rc (50 μM) with or without MLN-4760 (100 nM) for 24 h. **(A)** Western blot analysis for NOX2. **(B)** IF staining for NOX2. Magnification, ×200; scale bar = 200 μm. Data were expressed as mean ± SD from three independent experiments. ^##^ *p* < 0.01 compared with control, ^**^ *p* < 0.01 compared with HG, and ^&^ *p* < 0.05 compared with HG **+** ginsenoside Rc.

**FIGURE 10:**
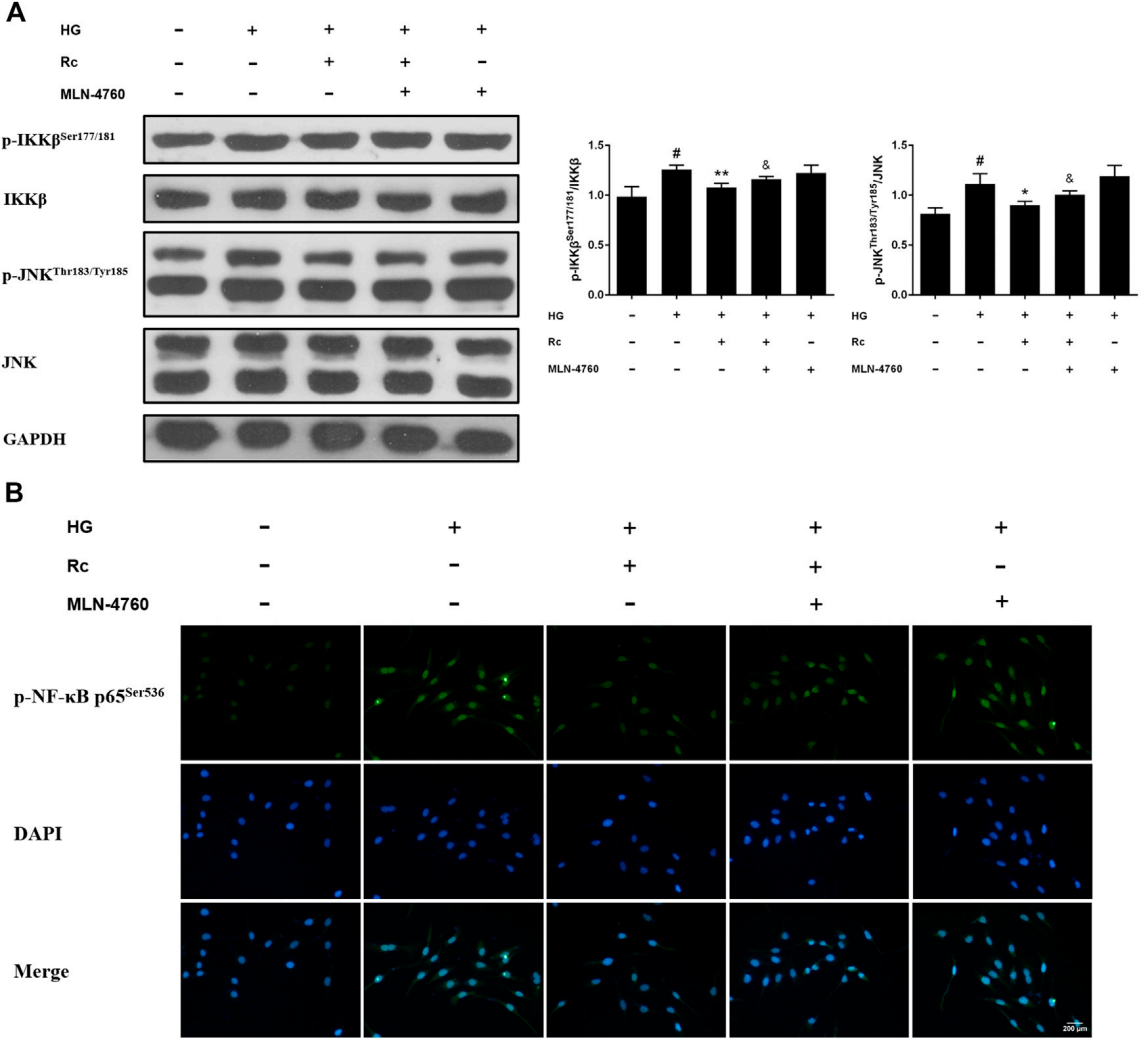
Effects of ginsenoside Rc with MLN-4760 on p-IKKβ^Ser177/181^ and p-JNK^Thr183/Tyr185^ phosphorylation in HUVECs followed by insulin (100 nM) stimulation for 30 min. The cells were treated with HG plus ginsenoside Rc (50 μM) with or without MLN-4760 (100 nM) for 24 h. **(A)** Western blot analysis for p-IKKβ^Ser177/181^ and p-JNK^Thr183/Tyr185^. **(B)** IF staining for p-NF-κB p65^Ser536^. Magnification, ×200; scale bar = 200 μm. Data were expressed as mean ± SD from three independent experiments. ^#^ *p* < 0.05 compared with control, ^*^ *p* < 0.05 and ^**^ *p* < 0.01 compared with HG, and ^&^ *p* < 0.05 compared with HG **+** ginsenoside Rc.

To further verify the ACE2-dependent mechanism of ginsenoside Rc, we evaluated the effects of MLN-4760 on protein expressions of NOX2 and inflammation pathways. Figures 9A,B showed that the inhibition of ACE2 by MLN-4760 markedly increased the level of NOX2. Besides, attenuated phosphorylation of IKKβ and JNK was partially reversed by MLN-4760 (Figure 10A). Similar results were observed in IF for p-NF-κB p65 (Figure 10B). MLN-4760 treatment alone did not regulate the proteins above. These data lead us to conclude that upregulation of ACE2 is indeed involved in the effects of ginsenoside Rc in HG-induced HUVECs.

### Ginsenoside Rc Upregulated ACE2 and Enhanced Degradation of Ang II Without Affecting Glycolipid Metabolic Parameters in db/db Mice

**FIGURE 11:**
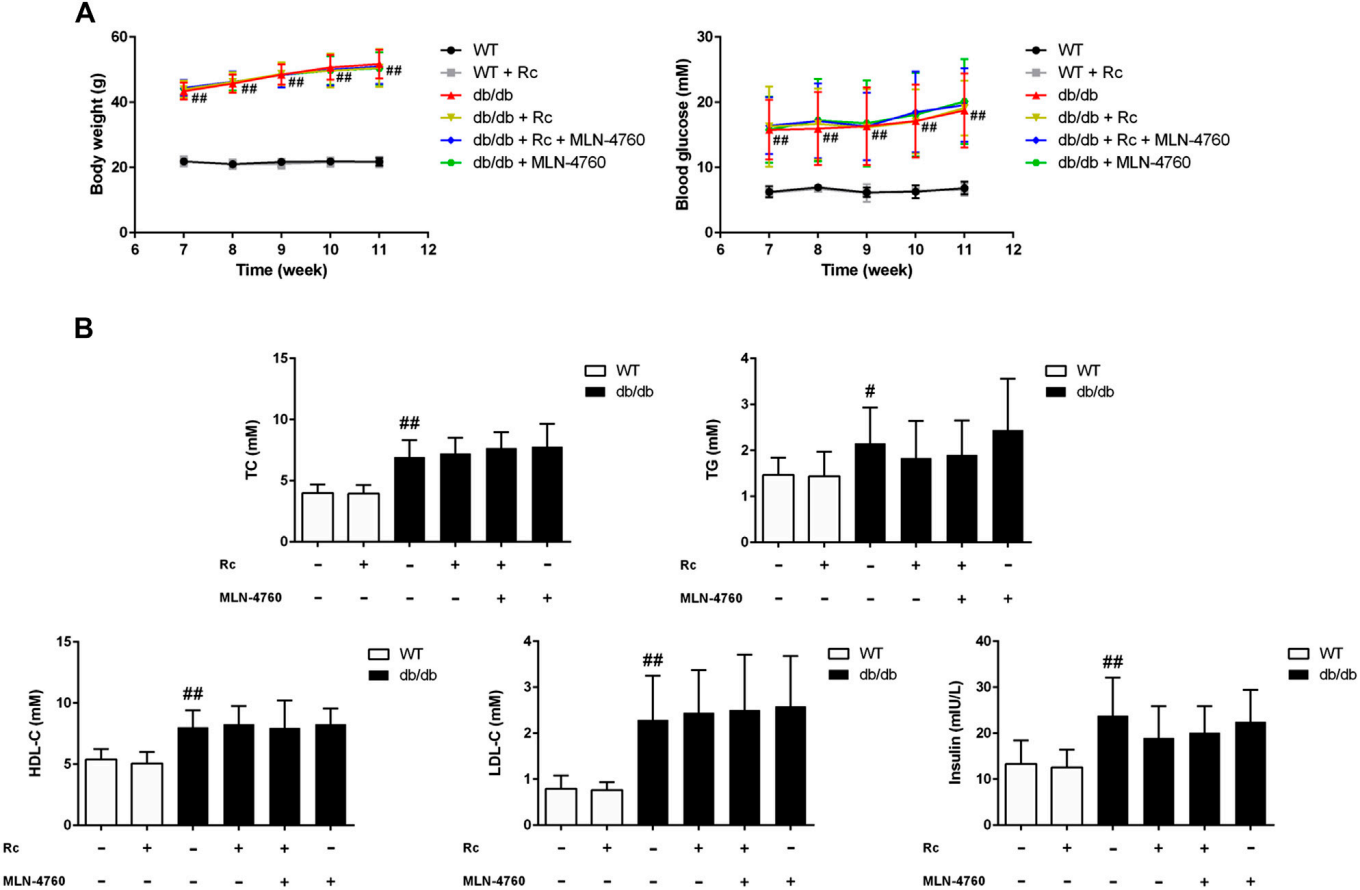
Effects of ginsenoside Rc on glycolipid metabolic parameters *in vivo.* **(A)** Body weight and FBG. **(B)** TC, TG, LDL-C, HDL-C, and insulin in serum. Data were expressed as mean ± SD (n = 10). ^#^ *p* < 0.05 and ^##^ *p* < 0.01 compared with WT.

**FIGURE 12:**
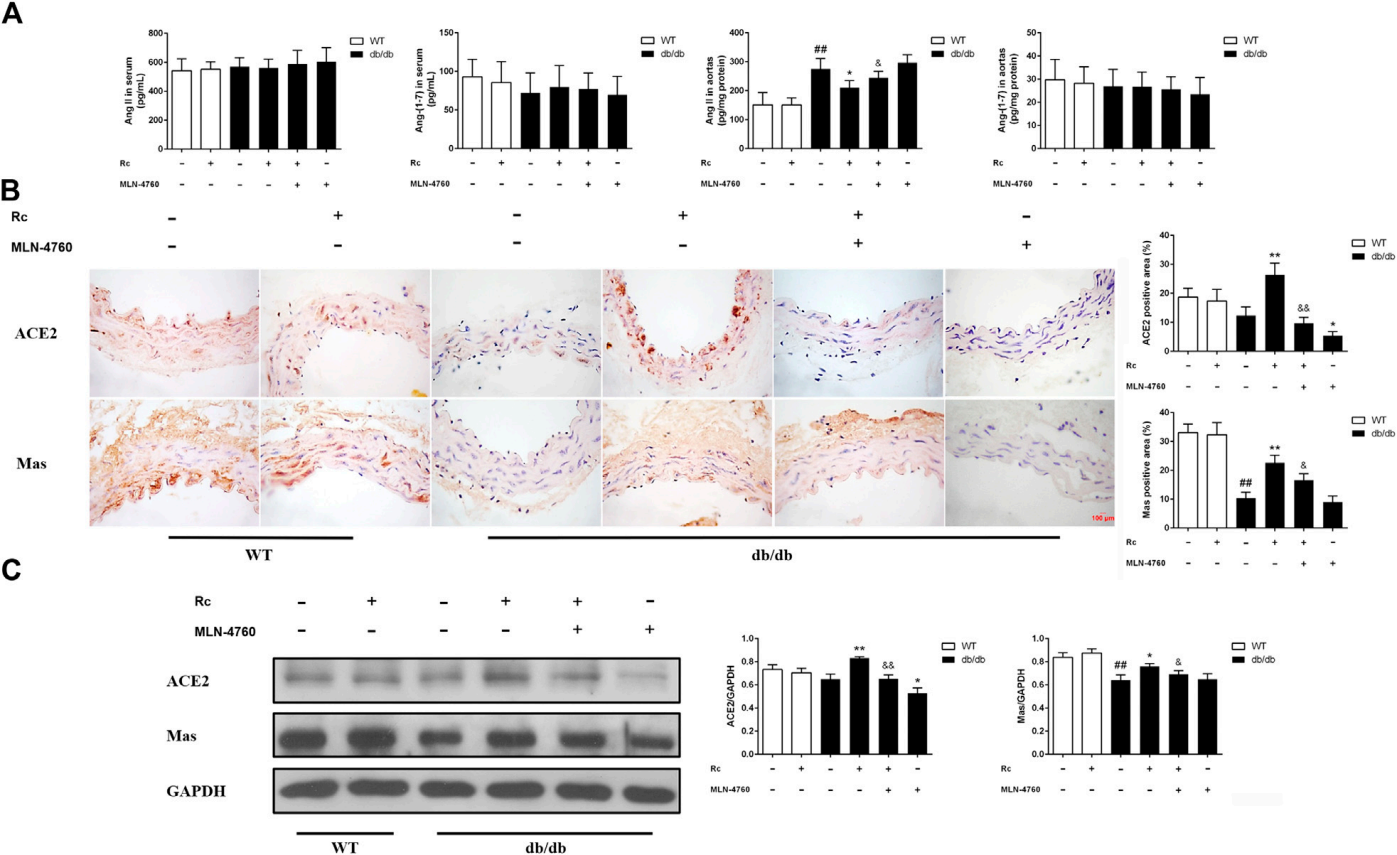
Effects of ginsenoside Rc on Ang II level and ACE2/Ang-(1–7)/Mas axis *in vivo.* **(A)** Ang II and Ang-(1–7) levels in serum (n = 10) and aorta tissue homogenate (n = 6). **(B)** IHC analysis for ACE2 and Mas in aortas (n = 3). **(C)** Western blot analysis for ACE2 and Mas in aortas (n = 3). Magnification, ×400; scale bar = 100 μm. Data were expressed as mean ± SD. ^##^ *p* < 0.01 compared with WT, ^*^ *p* < 0.05 and ^**^ *p* < 0.01 compared with db/db, and ^&^ *p* < 0.05 and ^&&^ *p* < 0.01 compared with db/db **+** ginsenoside Rc.

Based on the findings of our *in vitro* experiments, we next conducted *in vivo* studies applying db/db mice of the T2DM model. Meanwhile, MLN-4760 was put into use to determine the role of ACE2 in the effects of ginsenoside Rc *in vivo*. As was shown in Figure 11A, the body weight and FBG in db/db mice were much more higher than WT mice, which were not affected by either ginsenoside Rc or MLN-4760. Lipid profiles and insulin levels in db/db mice were strikingly higher than WT mice. Meanwhile, the insulin level in db/db mice treated with ginsenoside Rc presented a descending trend, which failed to reach significance (Figure 11B). Interestingly, there was a remarkable elevation of local Ang II production in aortas from db/db mice compared with WT mice, though Ang II levels in serum were similar among each group (Figure 12A). Ginsenoside Rc induced a significant decline of Ang II in aortas, which was reversed by MLN-4760. Moreover, we observed a reduction of Ang-(1–7) in db/db mouse serum without significance, which was accompanied by no significant change in aortic Ang-(1–7) level among each group. As was shown in Figure 12C, there was a declining trend in aortic ACE2 expression without an obvious difference, while Mas expression decreased significantly compared with WT mice. However, ginsenoside Rc significantly increased ACE2 and Mas expressions, accompanied by strong inhibitory effects of MLN-4760, which was consistent with the results of IHC (Figure 12B).

### Ginsenoside Rc Ameliorated Endothelial Dysfunction and Suppressed Inflammation As Well As Oxidative Stress in db/db Mice

**FIGURE 13:**
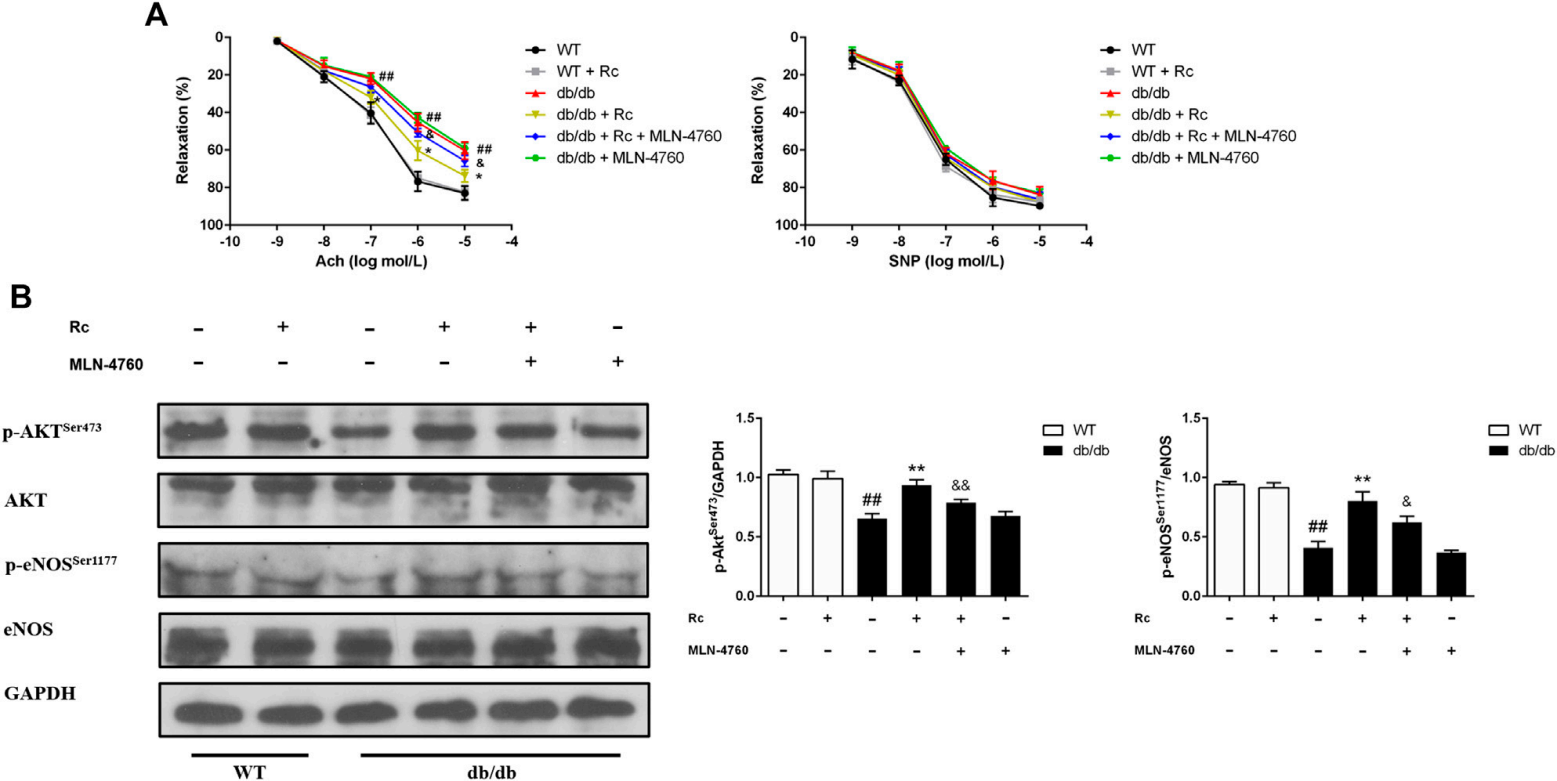
Effects of ginsenoside Rc on thoracic aortic relaxation *ex vivo* and aorta Akt/eNOS pathway *in vivo*. Dose-response relaxation was measured for cumulative increments of ACh (1 nM–10 μM) as well as SNP (1 nM–10 μM) after thoracic aortic rings were preconstricted with PE (1 μM). **(A)** Relaxation response to cumulative addition of ACh and SNP. **(B)** Western blot analysis for p-Akt^Ser473^ and p-eNOS^Ser1177^. Data were expressed as mean ± SD (n = 3). ^##^ *p* < 0.01 compared with WT, ^*^ *p* < 0.05 and ^**^ *p* < 0.01 compared with db/db, and ^&^ *p* < 0.05 and ^&&^ *p* < 0.01 compared with db/db + ginsenoside Rc.

**FIGURE 14:**
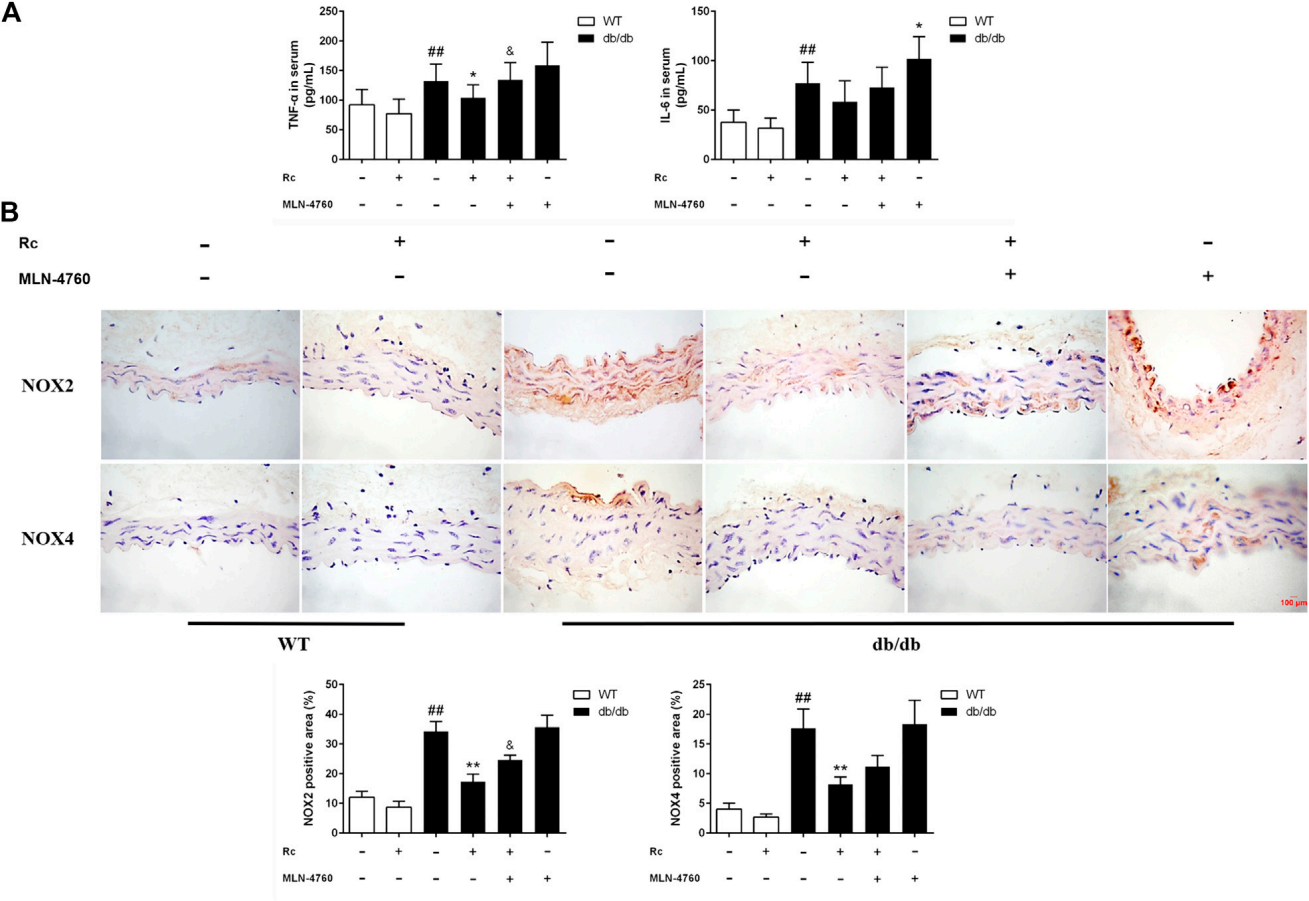
Effects of ginsenoside Rc on serum TNF-α and IL-6 levels, aorta NOX2, and NOX4 expressions *in vivo.* **(A)** TNF-α and IL-6 levels in serum (n = 10). **(B)** IHC analysis for NOX2 and NOX4 in aortas (n = 3). Magnification, ×400; scale bar = 100 μm. Data were expressed as mean ± SD. ^##^ *p* < 0.01 compared with WT, ^*^ *p* < 0.05 and ^**^ *p* < 0.01 compared with db/db, and ^&^ *p* < 0.05 compared with db/db **+** ginsenoside Rc.

To demonstrate whether ginsenoside Rc administration could improve endothelial dysfunction *in vivo*, we examined endothelium-dependent relaxation and Akt/eNOS pathway in aortas. As expected, the endothelium-dependent relaxant responses to ACh were significantly impaired in db/db mice compared with WT mice. However, ginsenoside Rc restored the impaired endothelium-dependent relaxation in aortas obtained from db/db mice, which was partly reversed by MLN-4760 (Figure 13A). Additionally, SNP-induced endothelium-independent relaxations were similar among each group (Figure 13A). The Western blot analysis showed that ginsenoside Rc dramatically promoted p-Akt and p-eNOS expression in aortas of db/db mice without affecting WT mice, whereas MLN-4760 markedly attenuated the effects above (Figure 13B). It is worth noting that ginsenoside Rc did not affect WT mice and MLN-4760 alone did not affect db/db mice. Next, we detected the levels of proinflammatory factors and NADPH oxidases. As was shown in Figure 14A, significant augmentation in the levels of TNF-α and IL-6 was found in db/db mouse serum. Ginsenoside Rc treatment induced a significant reduction of TNF-α, which was abolished by MLN-4760. However, the reduction in serum IL-6 induced by ginsenoside Rc did not achieve statistical significance and MLN-4760 alone further increased IL-6 level markedly. The IHC analysis demonstrated that the group treated with ginsenoside Rc produced a marked decrease in NOX2 and NOX4 expression compared with WT mice (Figure 14B). However, MLN-4760 partly inhibited the effects of ginsenoside Rc on reducing NOX2 and NOX4 expression. These results indicate that ginsenoside Rc ameliorates endothelial dysfunction and exhibits anti-inflammatory as well as antioxidant abilities through upregulating ACE2 *in vivo*.

**FIGURE 15:**
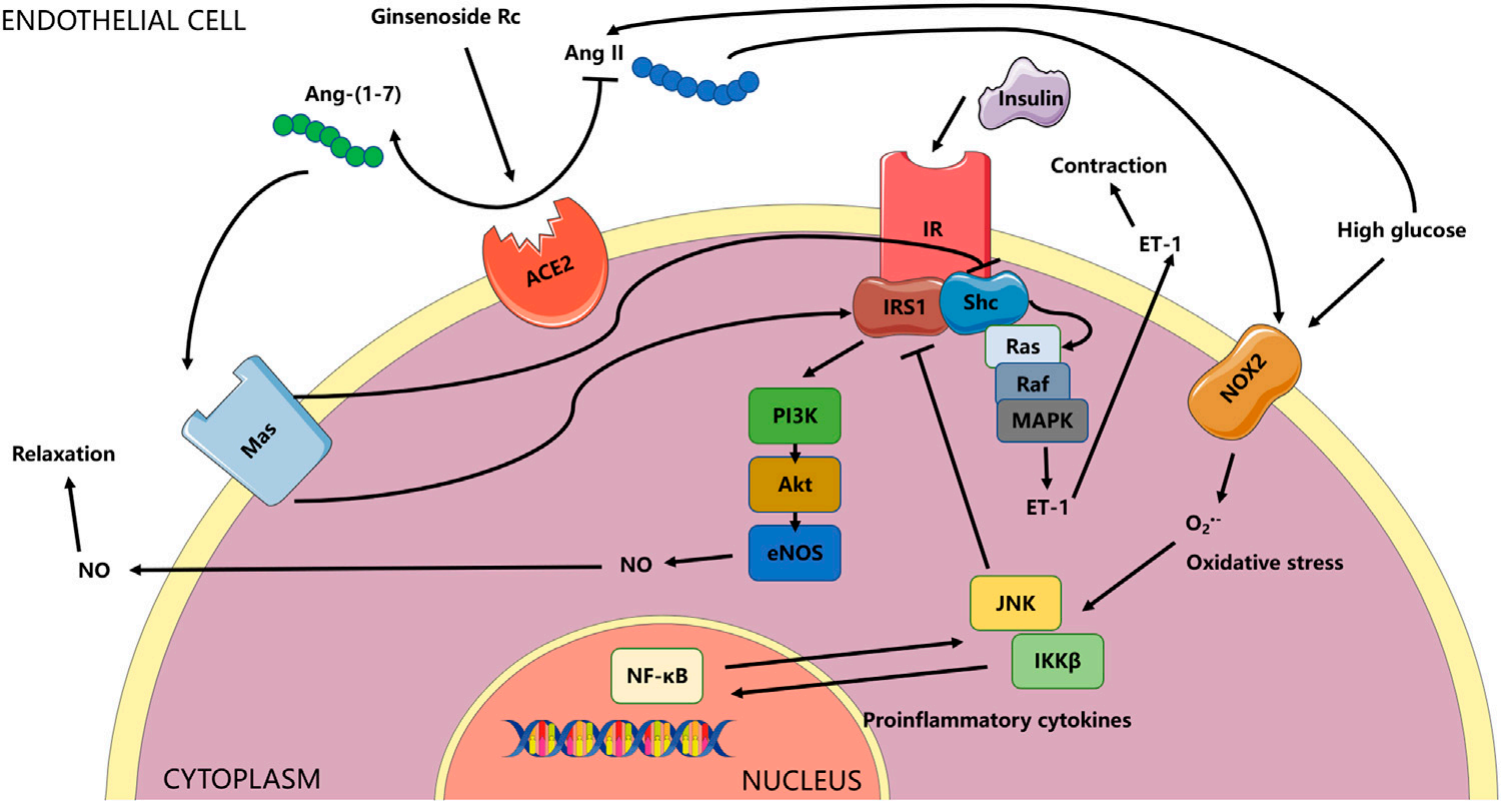
Schema for the current study. The effects of ginsenoside Rc on ameliorating endothelial IR and endothelial dysfunction relied on upregulation of ACE2 with subsequent restoration of insulin signaling related to antioxidation and anti-inflammation. The ACE/Ang II/AT_1_R axis could play a vital role in the activation of MAPK/ET-1 and NADPH oxidases, which was not explored in the current research and was not shown in the schema. Whether the effects of ginsenoside Rc are also through affecting the axis above or through other mechanisms deserves further study.

## Discussion

T2DM has become a major health concern, which has devastating effects on blood vessels, namely, microvascular and macrovascular complications, and vascular disease remains the leading cause of morbidity and mortality in individuals with diabetes (Sarwar et al., 2010; Patel et al., 2014; Vaidya et al., 2015). IR, regarded as the pathogenic hallmark of T2DM, is also present in cardiovascular diseases which are characterized by endothelial dysfunction (Kim et al., 2006; Muniyappa and Sowers, 2013). Endothelium is not only an impermeable inner covering of the blood vessels but also a paracrine, autocrine, and endocrine organ which plays a key role in homeostasis by secreting various vasoactive and trophic substances that affect vasomotion, endothelial and vascular smooth muscle cell growth and proliferation, endothelial-leukocyte interactions, platelet adhesion, coagulation, inflammation, and permeability (Pober and Sessa, 2007; Vanhoutte et al., 2009). Among the molecules secreted by endothelium, NO and ET-1 play a crucial part in modulating vascular tone and vessel integrity stimulated by insulin (Baratchi et al., 2017), which are often chosen as the markers of endothelial IR (Kearney et al., 2008). HG has been widely used to establish endothelial IR model in HUVECs (Yang et al., 2010; Ying et al., 2014; Kong et al., 2019). In the present study, HG treatment decreased NO content, elevated ET-1 mRNA level, caused significant augment of ROS, and produced impairment in insulin signaling pathway, suggesting a successful IR model in HUVECs. In addition, osmotic control (23 mM mannitol + 7 mM d-glucose) group showed that osmotic pressure in this study did not affect parameters tested in HUVECs (data not shown).

The balance between NO-dependent vasodilatation and ET-1-dependent vasoconstriction, which are mediated by insulin, is regulated by IRS-1/PI3K/Akt/eNOS and Ras/Raf/MEK/ERK pathways in the endothelium respectively (Nystrom and Quon, 1999; Saltiel and Kahn, 2001). Under insulin-resistant conditions where the pathway-specific impairment in PI3K-dependent signaling occurs, the imbalance between NO and ET-1 may result in endothelial dysfunction with subsequent cardiovascular events. Indeed, endothelial IR is typically accompanied by a reduced PI3K/NO pathway and an intact or heightened ERK/ET-1 pathway (Potenza et al., 2005; Hitomi et al., 2011; Muniyappa and Sowers, 2013). It is worth noting that elucidation of the insulin signaling pathway regulating endothelial production of NO reveals striking parallels with the metabolic insulin signaling pathway in skeletal muscle and adipose tissue (Montagnani and Quon, 2000; Vincent et al., 2003). Vascular actions of insulin to stimulate production of NO from endothelium contribute to increased blood flow that further enhances metabolic actions of insulin to promote glucose uptake in skeletal muscle (Baron and Clark, 1997; Vincent et al., 2004). Impairment in shared PI3K-dependent insulin signaling leads to reciprocal relationships between IR and endothelial dysfunction. Consequently, treatment aiming at improving endothelial IR is expected to exhibit beneficial effects on both metabolic and vascular functions simultaneously (Muniyappa et al., 2007). In the current study, we emphasized endothelial IR (changes in the insulin signaling pathway) as well as the effects of ACE2 on it; hence, each group of *in vitro* experiments was stimulated by insulin to make conditions identical. Indeed, the insulin control group (normal glucose without insulin stimulation) revealed that insulin treatment activated insulin signaling without affecting ACE2/Ang-(1–7)/Mas axis (data not shown). Our results showed that ginsenoside Rc not only corrected the imbalance between NO and ET-1 but also restored the IRS-1/PI3K/Akt/eNOS pathway.

Accumulating research has suggested that ACE2/Ang-(1–7)/Mas axis plays a vital role in the improvement of endothelial dysfunction (Rabelo et al., 2011; Fraga-Silva et al., 2013). Besides, it is well established that HG is able to induce Ang II production and reduce ACE2 expression in vascular smooth muscle cells (Lavrentyev et al., 2007; Lavrentyev and Malik, 2009), whereas little information is available in the literature on the effects of HG on the ACE2/Ang-(1–7)/Mas axis in HUVECs. Despite a study reporting that Ang-(1–7) could attenuate Ang II-induced impairment in insulin signaling (Tassone et al., 2013), further exploration into the molecular mechanisms underlying IR will be conducive to the prevention and treatment for diabetes and its complications. In the current study, it is interesting to find that ACE2 and Mas mRNA levels did not significantly differ between the NG and HG groups, while ACE2 mRNA level of vascular smooth muscle cells was markedly reduced after 24 h exposure to HG (Lavrentyev and Malik, 2009). It is possible that ACE2 may be altered in a tissue-specific manner at the level of ACE2 protein but remain unchanged at the gene level (Ye et al., 2004; Wysocki et al., 2006). The mechanism by which ACE2 or Mas protein is altered in the presence of untouched mRNA levels has not been investigated much, though posttranscriptional processing could explain these observations (Lambert et al., 2014). As for ginsenoside Rc, we observed that it managed to reduce the production of Ang II and activate ACE2/Ang-(1–7)/Mas axis with unchanged mRNA levels among each group. Therefore, in this study, ACE2 upregulation by ginsenoside Rc has been suggested as a therapeutic target in HG-induced HUVECs. To elucidate the possible mechanism on the effect of ginsenoside Rc on the amelioration of endothelial IR, we used a specific ACE2 inhibitor, MLN-4760, which exerts its inhibitory action by binding to two metallopeptidase catalytic subdomains of the ACE2 enzyme (Towler et al., 2004). Here, we showed that MLN-4760 partially reversed the effects of ginsenoside Rc on reducing Ang II and activating ACE2/Ang-(1–7)/Mas axis. In addition, ginsenoside Rc-induced improvement of endothelial IR was also abrogated by MLN-4760. It has been proved that Ang-(1–7) activated PI3K/Akt/eNOS pathway via Mas to exert beneficial effects (Sampaio et al., 2007). Similarly, the study of Rodrigo A et al. showed that ACE2 activator XNT could improve endothelia dysfunction, which was inhibited by Mas antagonist A779 or in Mas-knockout mice, suggesting the potential role of Ang-(1–7)/Mas pathway (Fraga-Silva et al., 2013). Given this, it is very likely that ginsenoside Rc improved the impaired ACE2/Ang-(1–7)/Mas axis with subsequent insulin signaling not only by the degradation of Ang II but also by the increase of Ang-(1–7). The results above suggested that the mechanism underlying the effects of ginsenoside Rc was dependent on the upregulation of ACE2, at least in part. However, further investigations are necessary to determine the impacts of ginsenoside Rc on ACE/AT_1_R/MAPK pathways, the role of Mas antagonist such as A779 in the effects of ginsenoside Rc, and how ginsenoside Rc upregulates ACE2.

Oxidative stress is a key factor leading to IR and endothelial dysfunction, where excessive ROS impair vasodilatation (Kim et al., 2006; Prieto et al., 2014). NO produced by eNOS may be inactivated by reaction with superoxide anions (O_2_^−^) to form peroxynitrite anion (OONO^−^), which reduces NO bioavailability (Moncada and Higgs, 2006). Emerging evidence has indicated that NADPH oxidase is a main source of ROS (Bayraktutan et al., 1998; Griendling et al., 2000), among which NOX2 and NOX4 are highly expressed in vascular endothelial cells (Konior et al., 2014). Previous studies have reported that inhibition of NOX2 alleviated endothelial dysfunction as well as endothelial and metabolic IR; meanwhile, HG stimulated ROS production by NOX2 (Sukumar et al., 2013; Souto Padron de Figueiredo et al., 2015). Moreover, Ang II whose production was promoted by HG has been shown to activate NADPH oxidase (Murdoch et al., 2011). ROS which could be augmented by hyperglycemia activate proinflammatory pathways including IKKβ and JNK which subsequently phosphorylate inhibitory sites such as Ser^307^ of IRS-1 (Ozes et al., 2001; Gao et al., 2002; Bloch-Damti and Bashan, 2005; Kamata et al., 2005). Moreover, IKKβ acts as the upstream signaling of NF-κB which stimulates the production of proinflammatory cytokines including TNF-α, IL-1β, and IL-6 which in turn further activate IKKβ and JNK (Ajuwon and Spurlock, 2005; Olefsky and Glass, 2010; Solinas and Karin, 2010; Macías-Pérez and Aldaba-Muruato, 2019). Accordingly, ways that are able to scavenge ROS or inhibit inflammation have the potential to protect against endothelial IR and endothelial dysfunction caused by various factors. In the current study, we observed that HG significantly increased NOX2 expression with NOX4 unchanged, which is also consistent with Yang Zhang’s research (Zhang et al., 2015), while ginsenoside Rc markedly suppressed NOX2 expression. Additionally, HG-induced IKKβ/NF-κB activation and JNK phosphorylation were remarkably inhibited by ginsenoside Rc. However, the effects of ginsenoside Rc above were reversed by MLN-4760 to a certain extent. Consequently, our results indicated that the effects of ginsenoside Rc in the amelioration of endothelial IR involved its prominent antioxidant and anti-inflammatory activities which could be partially ascribed to upregulation of ACE2.

The db/db mouse is a genetic model of T2DM caused by a spontaneous mutation of the leptin receptor gene that results in a shorter intracellular domain of the receptor with subsequent invalid signal transduction (Hummel et al., 1966; Chen et al., 1996). As a result of this mutation, hypothalamic responses to satiety are inhibited, which leads to hyperphagia and development of hyperglycemia accompanied by obesity and IR at about 4–7 weeks after birth (Hummel et al., 1966; Sharma et al., 2003). Diabetes is more severe in C57BLKS/J strain than C57BL/6 strain and more severe in male mice than female mice (Hummel et al., 1972; Kitada et al., 2016). Despite their limitations for the study of human T2DM, db/db mice are the most widely used model of T2DM (Tesch and Lim, 2011; Wang et al., 2014). In the present study, db/db mice were employed, and we discovered that ginsenoside Rc markedly increased aortic ACE2 and Mas expression in db/db mice, though it did not affect body weight and glycolipid metabolic parameters. However, the lack of oral glucose tolerance test rendered this study insufficient to demonstrate the effects of ginsenoside Rc on metabolic IR, which could be attributed to the improvement of endothelial IR or/and independent action on metabolic IR. Meanwhile, MLN-4760 prevented the increased expressions of ACE2 and Mas. It is well documented that Ang II plays an essential role in numerous pathophysiological processes resulting in increased ROS generation, enhanced inflammation, fibrosis, hypertrophy, and endothelial dysfunction (Matsusaka and Ichikawa, 1997; Muniyappa and Sowers, 2013). Our results revealed that repeated treatment of ginsenoside Rc in db/db mice significantly reduced local Ang II content in aortas with Ang-(1–7) content unchanged, indicating that the effects of ginsenoside Rc *in vivo* might rely more on the degradation of Ang II than the production of Ang-(1–7). Intriguingly, MLN-4760 alone remarkably aggravated the loss of ACE2 in aortas of db/db mice, which was distinct from the *in vitro* experiment. This could be on account of the different concentrations of MLN-4760 and the low level of ACE2 per se in the HG group *in vitro*. Besides, the reason why MLN-4760 alone did not affect Ang-(1–7) content significantly might be that there were alternative pathways for the production of Ang-(1–7) (Trask et al., 2010). It has been well described that the endothelium-dependent relaxations are impaired in various models of diabetes (Eckel et al., 2002). Our data showed that the endothelium-dependent relaxant responses to ACh were severely damaged in db/db mice, whereas the endothelium-independent vasodilation induced by SNP was not at all, suggesting a well-characterized model of endothelial dysfunction in T2DM. The impairment was alleviated by ginsenoside Rc, which was partly inhibited by MLN-4760. These results implied that dysfunction of endothelial cells occurred while the function of vascular smooth muscle cells remained intact in the early phase of T2DM. Additionally, repeated treatment of ginsenoside Rc improved the impaired Akt/eNOS pathway in db/db mouse aortas and reduced the production of proinflammatory cytokines in serum as well as NADPH oxidases in aortas from db/db mice, whereas MLN-4760 resulted in the attenuation of the effects of ginsenoside Rc above. In general, all these results indicated that ginsenoside Rc was capable of ameliorating endothelial dysfunction via upregulating ACE2 *in vivo*. However, like many natural compounds, ginsenoside Rc is a natural active product with relatively low potency, which is indeed a limitation for ginsenoside Rc requiring high doses to achieve efficacy.

In conclusion, despite more detailed mechanisms needed to be determined, our findings not merely reveal a novel action with its possible mechanism of ginsenoside Rc which effectively ameliorated endothelial IR and endothelial dysfunction, at least in part, via upregulation of ACE2 but also provide new insight into the potential clinical application of ginsenoside Rc which holds promise for the treatment of diabetic vascular complications.

## Data Availability

The datasets generated for this study can be found in the GeneBank containing ACE2 (NM_001386260.1), Mas (NM_001366704.2), ET-1 (NM_001168319.2), TNF-α (NM_000594.4), IL-1β (NM_000576.3), IL-6 (NM_001371096.1), GAPDH (NM_001357943.2).
